# Radiopharmaceutical administration practices—Are they best practice?

**DOI:** 10.3389/fnume.2023.1244660

**Published:** 2023-10-13

**Authors:** Stephen Harris, James R. Crowley, Nancy Warden

**Affiliations:** ^1^ Vascular Wellness Management Solutions, Cary, NC, United States; ^2^ Department of Molecular Imaging, Carilion Clinic, Roanoke, VA, United States

**Keywords:** administration quality, extravasations, infiltrations, best administration practices, injection quality

## Abstract

**Background:**

The nuclear medicine community has stated that they are using best practices to gain venous access and administer radiopharmaceuticals, and therefore do not contribute to extravasations. We tested this hypothesis qualitatively and quantitatively by evaluating four different perspectives of current radiopharmaceutical administration practices: (1) clinical observations of nuclear medicine technologists on the job, (2) quality improvement (QI) projects, (3) a high-level survey of current practices in 10 acute care hospitals, (4) intravenous (IV) access site data for 29,343 procedures. These four areas were compared to the gold standard of pharmaceutical administration techniques.

**Results:**

From clinical observations of radiopharmaceutical administrations in adult populations, technologists extensively used 24-gauge peripheral intravenous catheters (PIVCs) and butterfly needles. They also performed direct puncture (straight stick). Technologists predominantly chose veins in areas of flexion (hand, wrist, and antecubital fossa), rather than forearm vessels for IV access placement; in many circumstances, antecubital fossa vessels are chosen first, often without prior assessment for other suitable vessels. For selecting the injection vein, technologists sometimes used infrared vein finders but primarily performed blind sticks. Review of QI projects suggested that smaller gauge needles were contributing factors to extravasations. Additionally, the review of surveys from 10 hospitals revealed an absence of formalized protocols, training, knowledge, and skills necessary to ensure the safety/patency of IV devices prior to the administration of radiopharmaceuticals. Finally, findings from a review of IV access data for 29,343 procedures supported the observations described above.

**Conclusions:**

We expect that nuclear medicine technologists have the best intentions when providing patient care, but many do not follow venous access best practices; they lack formal protocols, have not received the latest comprehensive training, and do not use the best placement tools and monitoring equipment. Thus, the presumption that most nuclear medicine technologists use best practices may not be accurate. In order to improve radiopharmaceutical administration and patient care, the nuclear medicine community should update technical standards to address the most recent peripheral IV access and administration best practices, provide technologists with vascular visualization tools and the proper training, develop and require annual vascular access competency, and provide active monitoring with center and patient-specific data to create ongoing feedback.

## Introduction

In 2011, the American College of Radiology (ACR) and the Society of Nuclear Medicine (SNM) issued a Technical Standard for Diagnostic Procedures Using Radiopharmaceuticals^1^. While the Technical Standard does not specify the technique for vascular access during the administration, it does state the route of the administration must be verified prior to the administration of the radiopharmaceutical and that the amount of radioactivity and the route of administration must be recorded. Furthermore, the Technical Standard clearly states that there must be policies and procedures to ensure “…the radiopharmaceutical, the administered activity, and the route of administration are correct”. Additionally, because of the potential effects of ionizing radiation, the Technical Standard focuses on patient safety and the staff's responsibility to adhere to ALARA (as low as reasonably achievable) principles to minimize radiation exposure to patients. The Technical Standard was created because the proper administration of a diagnostic radiopharmaceutical is essential for radiation safety and nuclear medicine image quality and quantification.

An extravasation is the erroneous delivery of radiopharmaceutical into a patient's tissue instead of the venous system as intended. A diagnostic extravasation can compromise the quality and quantification of images, and thus sometimes the patient's ensuing care (1–6). It may also lead to unnecessary additional radiation doses for repeat imaging studies. Diagnostic extravasations can result in high absorbed dose to tissue (7), and there have been documented cases of deterministic effects (8).

Proper administration of a therapeutic radiopharmaceutical helps ensure the radiopharmaceutical reaches the target as prescribed. With the rapidly increasing use of radiotherapeutic applications, understanding and preventing extravasations have become increasingly critical. The higher radiation doses associated with therapeutic applications can lead to severe deterministic effects, posing potential harm to patients (8–10). While there is limited information available on diagnostic extravasations and their rates, literature on therapeutic extravasations remains even more limited. To the best of the authors' knowledge, no comprehensive studies specifically address radiotherapeutic extravasation rates.

Extravasations can result from issues associated with venous access and delivery of radiopharmaceuticals. Venous access can cause extravasations when inappropriate tools and/or lack of proper techniques create an unintended hole in the vein or result in missing the vein completely. Delivery can cause extravasations when catheter movements erode or puncture the vein or injection techniques “blow” the vein (high delivery pressure caused by small internal catheter diameter combined with rapid injection).

Over the past decade, nine studies from 13 centers have indicated that approximately 15% of radiopharmaceutical administrations are extravasated (11–17). While a recent single-center study reported an extravasation rate of less than 1%, this study did not calculate the true extravasation rate (18). Rather than using confirmed extravasations from a retrospective review of imaged injection sites or prospective monitoring, this study only captured extravasations that were noted in radiology reports, which grossly underestimates the true extravasation rate.

In areas of medicine using similar intravenous (IV) practices, the extravasation rate is significantly lower than in nuclear medicine. In a national benchmarking study of the chemotherapy extravasation rate, 739,832 patients were assessed. The extravasation rate was 0.09% (19). In 2015, a National Data Registry and Practice Quality Improvement Initiative involving 454,497 administration of non-ionic iodinated contrast medium found an extravasation rate of 0.24% (20). A single-center study conducted between 2008 and 2013 reported a combined 0.11% (541/502,391) extravasation rate for CT (0.13%) and MRI (0.06%) (21).

Concerted efforts to continually improve administrations resulted in lower extravasation rates than in nuclear medicine. It is likely these efforts exist because chemotherapy or CT extravasations are highly observable. Chemotherapy and CT contrast agent delivery typically involves large injection volumes and therefore extravasations can readily be seen due to changes in the overlying skin near the injection site. When these extravasations occur, patients see that the administration went wrong. In these cases, patients are typically informed they were extravasated, instructed on follow-up and monitoring of the extravasation site, the incident is documented in medical records, and these extravasations are often reported to the Chief Medical Officer as part of hospital reporting.

Radiopharmaceutical extravasations often go undetected by patients, technologists, and nuclear medicine physicians. Nuclear medicine procedures usually use small injection volumes of non-vesicant radiopharmaceuticals that do not cause immediate, visible changes to the overlying skin near the injection site, so neither the technologist nor patients is aware of an extravasation. And because there is typically no burning sensation with many of the pharmaceuticals used in nuclear medicine procedures, patients rarely know they have been extravasated. Nuclear medicine physicians are also often unaware of extravasations because during their clinical interpretation of the images, the injection sites are often outside of the imaging field of view (FOV). Furthermore, we are unaware of reporting systems even on a local level to encourage quality improvement. Without awareness, there is no incentive to address extravasations.

When extravasations are visible in images, there is evidence that they are rarely noted in the radiology report (22, 23). Furthermore, if an extravasation has been seen and noted in the radiology report, the information is rarely shared outside the department (24) or to a regulatory agency. Only 21 extravasations were documented in the FDA's adverse event system between the years 1968 and 2019 (25) and none are included in the US Nuclear Regulatory Commission (NRC) database of medical events. Additionally, none of the major accreditation organizations (e.g., The Joint Commission or Intersocietal Accreditation Commission) audit the quality of radiopharmaceutical administrations during accreditation processes.

While the NRC regulates the use of radiopharmaceuticals to protect patients from inadvertent radiation exposure, they do not require extravasation reporting. In May 1980, the NRC established misadministration reporting regulations to reduce inadvertent radiation exposure to patients, but exempted extravasations from event reporting based on belief that extravasations were “virtually impossible to avoid” (26). The extremely low extravasation rates in other IV administrations suggest the exemption is incorrect. In 2020, a petition for rulemaking was submitted to the NRC that challenged the exemption for reporting extravasations. In this process, NRC solicited public comments. Medical societies, leading members of the nuclear medicine and radiology communities, and individual technologists submitted 396 written or oral comments to the NRC^2^.

A common theme throughout the 396 comments was that technologists followed “best practices to achieve the highest quality of administration; therefore, RPE [radiopharmaceutical extravasation] rates cannot be improved”. Some examples of these public comments include: “If you are already using best practice then what can you do to correct the issue? Sometimes veins go bad”; “Radiology residents are well-trained on IV quality practices and extravasation concerns in general. [Extravasations] are already addressed through institutional processes, standards and best practices, technologist/personnel IV competency evaluation, and other quality assurance methods”; “The techniques employed are taught routinely in technology and nursing programs and used by any nuclear medicine technologist hundreds of times per year. Registered technologists undergo specific training and monitoring of their administration techniques, which are universal”.

For this work, experts from nuclear medicine and vascular access evaluated the claimed “best practices” using qualitative and quantitative methods and compared them to currently acceptable practices for intravenous therapies/procedures^3^.

## Methods

Ethics approval is not applicable for the described methods in this manuscript. Data reviewed included observations of nuclear medicine technologists' techniques, findings from previously published Quality Improvement projects that did not require ethics approval, a survey of procedures and protocols in 10 acute care hospitals, and a database review that does not include protected health information.

### Clinical observations

With over 48 combined years of experience, the authors have qualitatively observed/supervised hundreds of nuclear medicine technologists performing radiopharmaceutical administrations (e.g., site selection, vascular access, delivery, and post-procedure care).

### Quality improvement (QI) projects

Radiopharmaceutical administration QI projects used the Define, Measure, Analyze, Improve, and Control (DMAIC) methodology—a data driven approach to improve process effectiveness. The DMAIC methodology provides insight into factors that lead to radiopharmaceutical extravasations as well as proven interventions that lead to sustainable reduction in extravasation rates.

The authors have experience in leading and participating in four different radiopharmaceutical administration QI projects (27, 28).

### A high-level survey

Ten technologists from ten nuclear medicine departments were surveyed on current practices in vascular access and administration of radiopharmaceuticals.

The survey included several multiple-choice questions and one open-ended question (Table 1) to provide a high-level qualitative overview of nuclear medicine department procedures and processes.

**Table 1:** Current practices in vascular access and administration of radiopharmaceuticals.

Questions	Answers	Results
1. How many years have you been a practicing Nuclear Medicine Technologist?	1. 0–3 years	1. 20% (2/10)
	2. 4–7 years	2. 20% (2/10)
	3. 8–11 years	3. 20% (2/10)
	4. Over 12 years	4. 40% (4/10)
2. Which statement do you agree with the most, Obtaining IV access has changed because…	1. Patients’ veins have gotten easier to start IVs.	1. 0% (0/10)
	2. Patients’ veins have gotten more difficult to start IVs.	2. 10% (1/10)
	3. Really no change since I started.	3. 90% (9/10)
3. Which statement most closely matches your orientation to IV access at your job.	1. Preceptor orientation until they said I was independent to start an IV.	1. 10% (1/10)
	2. I completed a formal IV training class followed by precepted supervision.	2. 0% (0/10)
	3. I had previous experience so no training at my present facility.	3. 90% (9/10)
4. Radiopharmaceutical extravasation is not a problem because it causes very little harm to patients.	1. True	1. 30% (3/10)
	2. False	2. 70% (7/10)
5. Please check acceptable injection methods at your current facility.	1. New PIVC Only	1. 0% (0/20)
	2. Butterfly steel needle	2. 60% (6/20)
	3. Direct syringe injection	3. 50% (5/20)
	4. Preexisting PIVC	4. 90% (9/20)
6. Have you had additional training from your facility on IV access since your orientation?	1. Yes	1. 10% (1/10)
	2. No	2. 90% (9/10)
7. Do you require blood return in the IV prior to radiopharmaceutical injection?	1. Yes	1. 50% (5/10)
	2. No	2. 50% (5/10)
8. With difficult IV patients what resources are available to obtain access? Check all that apply.	1. Fellow nuclear medicine technologists only.	1. 60% (6/17)
	2. Ultrasound that Nuclear Medicine Techs use.	2. 0% (0/17)
	3. Vein visualization (Near infrared for example)	3. 40% (4/17)
	4. IV team availability	4. 70% (7/17)
	5. No other resources available	5. 0% (0/17)
9. Which of the following best describes your current facility?	1. There are written policies and procedures for IVs in my specific department.	1. 20% (2/10)
	2. There are not written policies and procedures for IVs in my department. It is part of our training.	2. 80% (8/10)
10. Which of the following best describes your current facility.	1. The facility maintains a yearly competency on my IV skill sets.	1. 10% (1/10)
	2. The facility does not require a yearly competency on my IV skill sets.	2. 90% (9/10)
11. What methods do you use to determine if the injection was successful? Please check all that apply	1. I feel the site during/after injection.	1. 80% (8/18)
	2. I ask the patient if it hurt at all.	2. 90% (9/18)
	3. There is no way to tell until scanned.	3. 10% (1/18)
	4. We have a device that monitors the injection.	4. 0% (0/18)
12. What method, if any, does your facility use to obtain consent for the nuclear medicine scan/injection.	1. Verbal consent from patient	1. 40% (4/10)
	2. Written consent from patient.	2. 20% (2/10)
	3. Consent is not required.	3. 40% (4/10)
13. What is your facilities policy if there is an inadvertent extravasation	1. Contact the Radiation Safety Officer.	1. 10% (1/14)
	2. Reschedule the exam.	2. 90% (9/14)
	3. Document in chart.	3. 10% (1/14)
	4. Cover the extravasation in a lead shield and attempt exam.	4. 10% (1/14)
	5. Re-Inject at another site.	5. 20% (2/14)

Survey questions and results.

The surveyed hospitals ranged in size from 61 to 1,032 beds. 5,495 hospital beds were represented in total, and the average size was 549 beds. The hospitals were located in the Midwest, Southeast, Northeast, and West to ensure a sample of hospitals across the United States.

### Database review

A database with information on 29,343 radiopharmaceutical administrations was searched to help characterize current radiopharmaceutical administration techniques. Searches included keywords such as “technique”, “needle”, and “location”. Key data, such as the number of procedures, IV site distribution, and needle gauge usage were extracted and analyzed using pivot tables to generate our findings.

### Gold standard of infusion techniques

*Infusion Therapy Standards of Practice (8th edition) (*the *Standards)* was referenced as the gold standard of infusion techniques. The *Standards* provide guidelines and clinical practice recommendations based on the most current evidence available. Standards are declarative statements, an expectation of the profession by which the quality of practice, service, or education is judged. They describe the action needed to provide competent care. The eleven highly credentialed vascular access committee members responsible for reviewing the Standards consulted over 2,500 literature sources to compile this edition. The final document was also peer-reviewed by a panel of 118 individuals from 17 countries and diverse health care specialties.

## Results

### Clinical observations

From clinical observations of radiopharmaceutical administrations in adult populations, technologists extensively used 24-gauge PIVCs and butterfly needles. They also performed direct puncture (straight stick). Technologists predominantly chose veins in areas of flexion (hand, wrist, and antecubital fossa), rather than forearm vessels for IV access placement; in many circumstances, antecubital fossa vessels are chosen first, often without prior assessment for other suitable vessels. For selecting the injection vein, technologists sometimes used infrared vein finders but primarily performed blind sticks. Additionally, technologists were often allowed to use pre-existing IVs.

### Quality improvement (QI) projects

One ^18F^FDG QI Project reported 459 injections were monitored during the 11-week study period. The extravasation rate was 12.8%. After educational intervention was implemented, including more thorough vein evaluation and ceasing use of butterflies and straight sticks, the extravasation rate decreased^4^.

A ^99m^TC-MDP QI Project reported a total of 816 administrations were studied, and contributing factors analysis revealed that injection site location, venous access technique, and needle gauge were associated with higher extravasation rates during the Measure Phase. Specifically, straight stick technique was associated with higher predicted probability of extravasation when compared to IV technique (*p* = 0.0001). And 23-gauge needle was associated with higher predicted probability of extravasation when compared to 22-gauge needle (*p* = 0.0001). Further analyses indicated that extravasation rates decreased from 12.75% to 3.4% when technologists reduced or discontinued the use of straight sticks and 23-gauge needles for venous access (28).

### High-level survey

The survey of technologists working in the nuclear medicine department in ten hospitals across the United States revealed an absence of formalized training, knowledge, and skills necessary to ensure the safety/patency of IV devices prior to the administration of radiopharmaceuticals. For example, no nuclear medicine department used current best practices as described in the *Standards*. None followed formalized vascular access training programs or required ongoing education for all clinicians administering radiopharmaceuticals. No nuclear medicine department documented radiopharmaceutical extravasations or notified the referring provider (Table 1).

Additionally, the survey findings revealed that technologists were not required to check for blood return in PIVCs after gaining venous access and before injecting.

### Database review

Findings from systematic review of IV access data for 29,343 procedures supported the observations described above. Forearm IV sites represented 7.9% of all sites and antecubital fossa represented 67.3%. 23–25-gauge PIVCs use represented 35.21% of all administrations.

### Gold standard of infusion techniques

#### Injection site location

Numerous recommendations within the *Standards* advocate utilization of forearm vessels and avoidance of areas of flexion such as hand, wrist, and antecubital fossa (Table 2).

**Table 2:** Infusion therapy standards of practice: guidelines and Recommendations on injection site location, vein selection and addressing extravasations.

Injection site location
“Avoid PIVC insertion in areas of: a. Flexion”.
“PIVC insertion in areas of flexion such as the hand is associated with higher rates of failure over time”.
“Short PIVCs inserted for infusion into veins of the antecubital fossa are not recommended due to higher catheter complication rates in areas of joint flexion”.
“Neonates and pediatric patients a. Avoid the antecubital fossa, which has a higher failure rate”.
“Assess the risk of mechanical causes of infiltration/ extravasation, which include i. PIVC sites in the hand, wrist, upper arm, foot, ankle, and antecubital fossa, when compared to sites in the forearm; inadequate catheter securement and joint stabilization if forced to use a site in an area of joint flexion”
Vein selection
“Use vascular visualization technology (e.g., near infrared, ultrasound) to increase success for patients with difficult intravenous access”.
“Use ultrasound to identify and assess vasculature, including: size, depth, and trajectory of vessels; anatomy to avoid, such as arteries and nerves; optimal site for PICC insertion; and to increase first-time insertion success”.
“Use ultrasound guidance for arterial puncture and catheter insertion in adults and children”.
“Vascular visualization technology is employed to increase insertion success of the most appropriate, least invasive vascular access device (VAD), minimizing the need to escalate to an unnecessary, more invasive device and to reduce insertion-related complications”.
Addressing extravasations
“Limit the extent of injury through early recognition of signs and symptoms of infiltration/extravasation”.
“Review infiltration/extravasation incidents causing harm or injury, using adverse event reports and health record reviews for quality improvement opportunities”.
“Investigate serious adverse events immediately to ensure prompt action and improve safety. The process includes a root cause analysis (RCA) or other systematic investigation and analysis to improve quality and safety”.
“Include patients in adverse event review when appropriate–

In current practices, areas of flexion are used in 92.1% of all cases from the database review. This is confirmed by our experience with QI projects and clinical observations. From the database review, antecubital fossa represented 67.3% and hand and wrist—18.8% of all site locations. In one of the QI projects, antecubital fossa represented 71.8% and hand and wrist—22.2% of site locations (28).

In nuclear medicine, forearm injection sites are seldom used for delivery of radiopharmaceuticals.

#### Vein selection

The *Standards* repeatedly emphasize the advantages of employing vein visualization for the identification and evaluation of vascular insertion sites (Table 2).

In current practices as confirmed by clinical observation, technologists sometimes used infrared vein finders but primarily performed blind sticks for selecting the injection vein. In the survey, no respondents reported using ultrasound, while four of ten of the technologists reported that near-infrared technology was available for use at their facility.

#### Addressing extravasations

The *Standards* stress the importance of promptly identifying signs and symptoms of extravasation to minimize injury, conducting an immediate investigation, and notifying patients (Table 2).

In our study of the 10 facilities and our clinical observations, we found that nuclear medicine departments rarely establish mitigation protocols or notify patients.

## Discussion

A significant gap exists between vascular access and delivery practices in nuclear medicine and practices in other areas of medicine. Several factors contribute to this gap. Nuclear medicine technologists traditionally do not receive specific training for vascular access. The survey and clinical observations found that vascular access knowledge is passed through on the job training when a new technologist joins the workforce. This on the job training does not always include the latest research on vascular access techniques. Additionally, technologists have no reason to suspect that they are not meeting the highest standards of injection quality since traditional monitoring approaches do not routinely provide consistent feedback. Also, a lack of ongoing vascular access competency contributes to the gap between best and actual practices.

Awareness and recognition of the problem is the first crucial step towards improving injection quality. Technologist education plans should be developed in collaboration with vascular access experts. These plans should include hands-on training in best practices for peripheral IV access and administration as well as continued annual competency assessments. In addition, nuclear medicine departments should add vascular visualization technologies and tools to their standard equipment.

The lack of feedback during administration is another area to address. Active monitoring of injection quality can provide immediate feedback to technologists and extravasation reporting can incentivize technologists to maintain a high level of the skills due to the observer effect (28). Additionally, recognition and other positive reinforcement can help motivate technologists to improve and then maintain a high level performance.

While there are commonalities across administrations of all pharmaceuticals, such as venous access techniques, there are some differences that are potential limitations of our findings. There are unique characteristics of radiopharmaceutical administrations, such as syringe shielding and rapid bolus injections of smaller volumes as compared to the typical administrations in CT, MR, and chemotherapy. Therefore, while these unique characteristics may contribute to the higher extravasation rate in nuclear medicine, they do not account for the magnitude of difference between nuclear medicine and other areas. If these characteristics were completely responsible for high extravasation rate in nuclear medicine, then we would expect to see consistently high rates across all technologists and centers. However, published rates in nine different centers in Alberta varied from 0%–44% (17). Other published studies show radiopharmaceutical rates can vary widely across different nuclear medicine facilities, ranging from 2% to 38% (16, 27). Additionally, in the largest radiopharmaceutical administration QI project 56 technologists participated. Their extravasation rates varied from 0% to 24.4% (27).

Our survey and clinical observations also are a limitation of our study. While survey findings and clinical observations regarding mitigation protocols and notifying patients are from a cross section of facilities and nuclear medicine departments, it would be necessary to conduct a much larger survey to generalize these practices across all of nuclear medicine.

## Conclusions

Our findings clearly suggest that many nuclear medicine professionals, even with the best intentions to provide high quality care, have not been provided the opportunity to use best practices to administer radiopharmaceuticals. Many technologists have not received the latest comprehensive training on venous access, do not use or do not have access to the best placement tools, and do not use prospective monitoring equipment. Additionally, technologists lack formal protocols, which is especially concerning in cases involving the administration of radioactive materials.

The QI projects highlighted in the study underscore the profound impact of using best practices, as indicated by the significant reduction in extravasation rates. If there were universal adoption of best practices, it's conceivable that such reductions in extravasation rates could become standard across the industry, greatly enhancing patient safety and the overall quality of care in nuclear medicine.

Several steps are needed to improve radiopharmaceutical administration quality; these include updating technical standards to address peripheral IV access and administration, providing technologists with vascular visualization tools and the proper training, developing and requiring annual vascular access competency, and providing active monitoring with center and patient-specific data to create ongoing feedback.

## Data Availability

The original contributions presented in the study are included in the article/Supplementary Material, further inquiries can be directed to the corresponding author.

## Footnotes

^1^ https://www.iaea.org/resources/guidelines/acr-snmmi-technical-standard-for-diagnostic-procedures-using-radiopharmaceuticals-2011

^2^ https://www.regulations.gov/docket/NRC-2020-0141/comments

^3^ https://journals.lww.com/journalofinfusionnursing/Citation/2021/01001/Infusion_Therapy_Standards_of_Practice,_8th.1.aspx

^4^ https://lucerno.com/wp-content/uploads/2023/02/WFU_Poster.pdf
